# The Dynamic Fungal Genome: Polyploidy, Aneuploidy and Copy Number Variation in Response to Stress

**DOI:** 10.1146/annurev-micro-041320-112443

**Published:** 2023-06-12

**Authors:** Pétra Vande Zande, Xin Zhou, Anna Selmecki

**Affiliations:** Department of Microbiology and Immunology, University of Minnesota, Minneapolis, Minnesota, USA;

**Keywords:** polyploidy, aneuploidy, copy number variation, genome plasticity, adaptation to stress

## Abstract

Fungal species have dynamic genomes and often exhibit genomic plasticity in response to stress. This genome plasticity often comes with phenotypic consequences that affect fitness and resistance to stress. Fungal pathogens exhibit genome plasticity in both clinical and agricultural settings and often during adaptation to antifungal drugs, posing significant challenges to human health. Therefore, it is important to understand the rates, mechanisms, and impact of large genomic changes. This review addresses the prevalence of polyploidy, aneuploidy, and copy number variation across diverse fungal species, with special attention to prominent fungal pathogens and model species. We also explore the relationship between environmental stress and rates of genomic changes and highlight the mechanisms underlying genotypic and phenotypic changes. A comprehensive understanding of these dynamic fungal genomes is needed to identify novel solutions for the increase in antifungal drug resistance.

## INTRODUCTION

**Figure 1:**
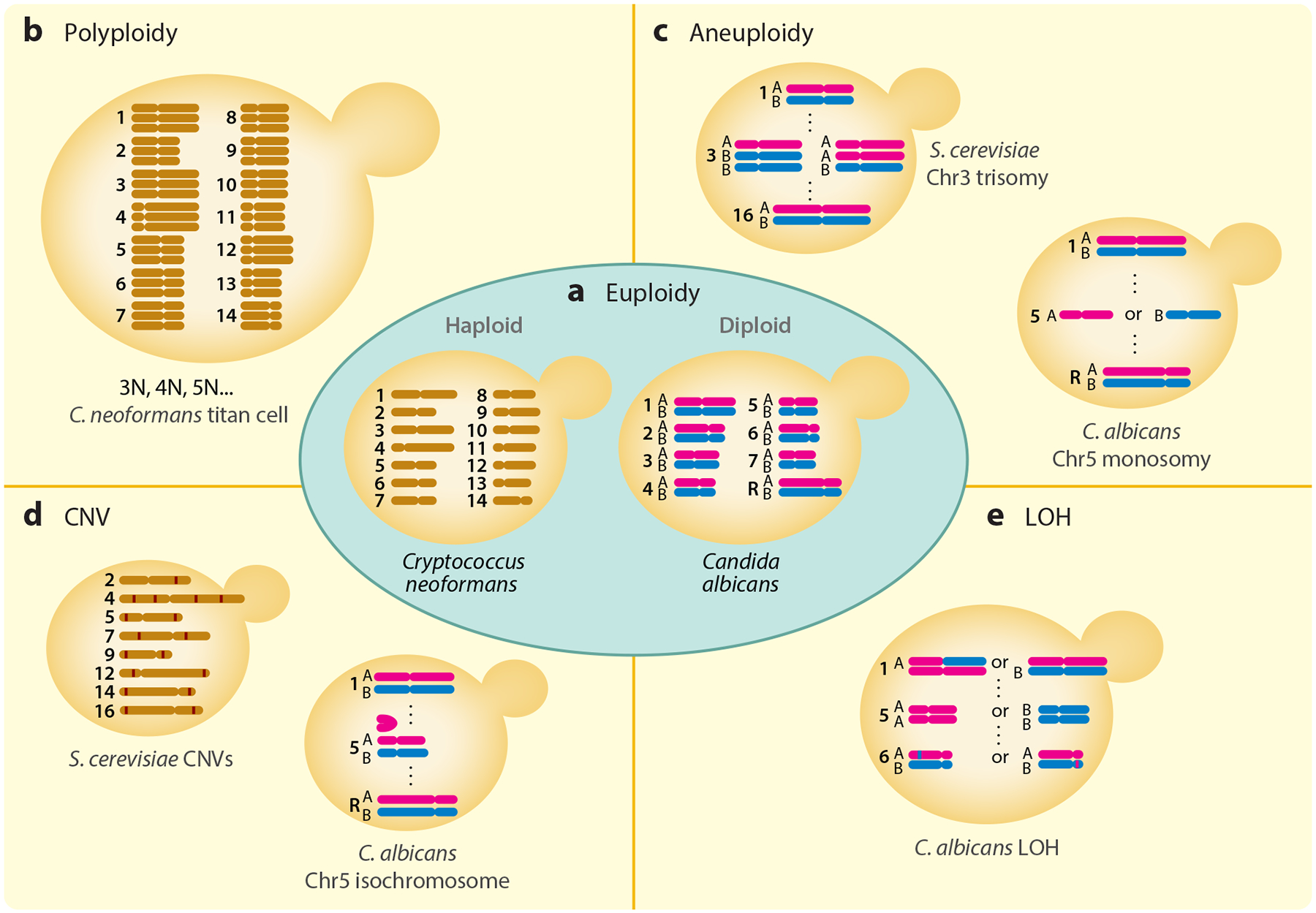
Examples of fungal cells exhibiting genomic changes. (*a*) Two representative euploid species: haploid (1N) *Cryptococcus neoformans* and diploid (2N) *Candida albicans*. (*b*) An example of polyploidy: *C. neoformans* titan cells contain at least 3 copies of the entire genome (3N and higher). (*c*) Examples of aneuploidy: *Saccharomyces cerevisiae* chromosome 3 trisomy and *C. albicans* chromosome 5 monosomy. (*d*) Example representation of many short intrachromosomal copy number variations (CNVs) in *S. cerevisiae* (red lines indicate increased copy number of that chromosomal region) and *C. albicans* isochromosome 5L. (*e*) An example of loss-of-heterozygosity (LOH) events in *C. albicans* includes short-tract LOH, long-tract LOH, and whole-chromosome LOH. Figure adapted from images created in BioRender.com.

Fungal pathogens in both clinical and agricultural settings are of increasing concern around the world. Their status as eukaryotes makes them particularly difficult to combat without detrimentally affecting their eukaryotic hosts, limiting the arsenal of effective antifungal drugs. This limited supply of antifungals is further threatened by the increasing acquisition of resistance often linked to large-scale genomic changes. Fungi exhibit a remarkable level of genomic plasticity (Figure 1), including changes in ploidy, aneuploidy, copy number variation (CNV), and loss of heterozygosity (LOH). Each of these genotypic changes can have a myriad of phenotypic consequences that in some cases allow the cell to adapt to environmental challenges, including nutrient limitation and antifungal drugs. In this review, we address the frequencies and rates of these genomic changes among environmental and clinical isolates of diverse fungal species. We also discuss the underlying mechanisms leading to these genomic changes and the consequences of those changes for fitness and gene expression phenotypes (26, 58, 112, 113, 120). We pay special attention to prominent fungal pathogens and model species.

From the mechanisms of genomic rearrangements that have been elucidated thus far, it is clear that environmental stress can increase the rate of genomic change, which further contributes to adaptation (19, 60, 82). In laboratory strains, aneuploidies in particular have been shown to carry a fitness cost (55, 87, 94, 131, 142, 151), but these costs are less pronounced and often absent in wild strains (58, 67, 109, 115). Changes in gene expression induced by aneuploidies and CNVs can have fitness advantages in particular environments that outweigh any disadvantage, potentially producing quick solutions to environmental challenges that allow species to thrive in new environments while continuing to explore the mutational space (33, 93, 133). In some cases, such as in yeasts used for industrial purposes, adaptation to new environments is desirable and genomic flexibility is encouraged. However, genomic changes resulting in unchecked growth and drug resistance in pathogenic fungi and tumor cells alike present life-threatening challenges for which we have limited solutions. A better understanding of fungal genome dynamics will improve our ability to better engineer industrial strains and prevent the development of antifungal resistance.

## THE FREQUENCY OF POLYPLOIDY, ANEUPLOIDY, AND COPY NUMBER VARIATION IN COMMON FUNGAL SPECIES

Genomic changes are widespread across environmental isolates of model fungal species (23, 73, 112, 113, 133, 151). Variation in ploidy is evident across 1,011 environmental isolates of *Saccharomyces cerevisiae*, where 1% are haploid (1N), 87% are diploid (2N), and 12% are polyploid (3N–5N) (97). The majority of these isolates are euploid, but 19% contain one or more aneuploid chromosomes, and the frequency of aneuploidy varies across lineages and ecological groups (70, 97, 109).

Variation in chromosome copy number is also prevalent in human pathogenic fungi, especially within the *Candida* genus. The opportunistic fungal pathogen *Candida albicans* accounts for a majority of all hospital-acquired fungal infections. While clinical isolates of *C. albicans* are predominantly diploid, rare haploid and polyploid isolates have been identified (39, 53), and the frequency of aneuploidy across all isolates varies from 5.4% to greater than 30% (39, 55, 104, 111, 112). The variation in aneuploid frequency across data sets may be attributable to either the frequency of isolates obtained from patients with prior exposure to antifungal drugs or the loss of aneuploidy that can occur during laboratory culture (11, 128, 129).

*Candida glabrata* and *Candida auris* are haploid species of significant medical concern, due to their intrinsic and acquired multidrug resistance. Clinical isolates from both species exhibit significant chromosome size differences by karyotype gel electrophoresis, but whole-genome ploidy changes are extremely rare (17, 65, 84, 98). In *C. glabrata*, 4 of 33 clinical isolates are aneuploid and carry extensive CNVs (17). In *C. auris*, aneuploidy, CNVs, and large chromosomal inversions occur between different clinical isolates and can arise in vitro with minimal passaging (12, 15, 85). The subtelomeric regions of *C. auris* contain extensive CNVs, predominantly deletions of many genes, which likely cause both karyotype and phenotype differences between isolates (15).

The presence of genomic variation in environmental and clinical isolates signifies that there is a nontrivial amount of standing genetic variation of these fungal populations. This standing genetic variation is the result of mutational input and natural selection acting upon variants. To understand how these two factors contribute to standing genetic variation in natural populations, we must understand the rates at which genomic changes arise.

## THE RATES OF GENOMIC CHANGE

To determine the rates of genomic change, several quantitative methods have been employed in different fungal systems. The most unbiased method of studying the rate and spectrum of genome-wide spontaneous mutations in the absence of selection is mutation accumulation (MA) experiments followed by whole-genome sequencing. MA lines pass through a single-colony bottleneck for many generations, enabling most mutations, even mildly deleterious mutations, to be quantified. In *S. cerevisiae*, MA experiments have been performed for 1,700 to 4,800 cell divisions in order to estimate mutation rates across different ploidy levels (30, 116, 151). On average, aneuploidy, CNV, and LOH occur at much higher rates (~1 × 10^−2^ to 1 × 10^−5^ events per diploid events per diploid genome per cell division) than the single-nucleotide mutation rate (~1 × 10^−10^ genome per cell division) than the single-nucleotide mutation rate (~1 × 10^−10^ per site per diploid genome per cell division) (29, 116, 151). In diploid MA lines, aneuploidy occurs at ~1 × 10^−4^ per division, which is more than 10 times higher than CNVs (29, 116, 151). These large changes due to aneuploidy and CNV occurred only in the diploid and polyploid MA lines, not in haploid MA lines (30, 116, 151), and the rate of LOH also increased dramatically with increasing ploidy: 9.3 × 10^−3^, 2.2 × 10^−2^, and 8.4 × 10^−2^ events per division in diploid, triploid, and tetraploid cells (30).

In diploid *C. albicans*, heterozygous counterselectable markers including *GAL1* and *URA3* have been used to determine rates of LOH at unique loci across each chromosome and across different environments. In the absence of stress, *C. albicans* LOH rates range from ~10^−6^ to ~10^−7^ events per diploid genome per cell division. However, in the presence of stress, including oxidative and antifungal stress, LOH rates increase greater than 72-fold (34). Additionally, fluorescent reporter systems in *C. albicans* have been used to determine the rates of LOH in a high-throughput manner (76). These reporter systems use strains with two genes encoding different fluorescent proteins at alleles of the same locus and analysis by flow cytometry to quantify the rate of fluorophore loss. The use of this method revealed that high temperature and DNA damage stress increased LOH rates by ~1.5-fold and >12-fold compared with no stress (76). Similar fluorescent reporter systems have been used to detect the frequency and dynamics of CNV formation in *S. cerevisiae* populations during adaptive evolution (5, 69). Individual cells with unique fluorescence phenotypes are isolated using fluorescence-activated cell sorting and then whole-genome sequenced, revealing the underlying genotypes and mechanisms driving CNV and LOH events. These methods highlight the potential of applying fluorescent reporters to further our understanding of the rate, dynamics, and mechanisms that underlie large-scale genome changes in diverse fungal species.

## MECHANISMS CAUSING POLYPLOIDY, ANEUPLOIDY, AND COPY NUMBER VARIATION

Whole-genome duplication events can arise both in vitro and in vivo through various mechanisms, including mating, endoreduplication, mitotic slippage, and cytokinesis failure (1, 49, 82). Typical mating between fungal cells with opposite mating types temporarily increases ploidy, followed by ploidy reduction via meiosis; in contrast, whole-genome duplication events occur in the absence of meiosis. For example, mating between different haploid serotypes of *Cryptococcus neoformans* (*MATa* serotype A and *MATα* serotype D) results in diploid AD hybrids. Some AD hybrids have a high frequency of aneuploidy that is a result of missegregation during meiosis between these genetically divergent (~10–15%) serotypes (6, 71, 73). Diploid *C. neoformans* cells also arise from unisexual mating, especially between *MATα* and *MATα* genotypes (72). *C. neoformans* can also undergo asexual polyploidization during infection of the human host that results in an amplification of genome copy numbers from 1N to 4N–128N (89, 144). These large, polyploid “titan” cells are mononucleated, indicating that they are formed by endoreduplication and cytokinesis failure (145, 147). Titan cells can be generated in vitro with host-relevant stimuli including serum, bronchial-alveolar lavage fluid, and a cell wall component from human lung–resident bacteria (24, 56, 132). Once titan cells form, they are genetically unstable and give rise to progeny that are highly aneuploid, in both the presence and the absence of selection (45).

In diploid *C. albicans*, tetraploid cells can form via mating of two cells with opposite mating types. These tetraploid cells do not undergo conventional meiosis or sporulation but instead undergo a parasexual process involving concerted chromosome loss and variable levels of recombination to a diploid or near-diploid level (3, 7, 35). This parasexual process generates aneuploid intermediates with different aneuploid chromosome combinations and adaptive potential. Similar nonmeiotic processes are observed in other fungi, parasites, and mammalian cells and act as important drivers of ploidy change, aneuploidy, and the generation of recombinant progeny (107). *C. albicans* can also undergo asexual ploidy changes during adaptation to stress, and rare clinical isolates are haploid or polyploid (53, 55). For example, upon exposure to an antifungal drug, *C. albicans* rapidly goes through whole-genome duplication events that occur through an ordered series of abnormal cell cycle events involving cytokinesis failure and the formation of binucleate tetraploid progeny that are capable of undergoing mitosis with multiple spindles, leading to unequal nuclear segregation and aneuploidy (50). In summary, ploidy changes in fungi, especially polyploidization, are associated with increased frequencies of additional large genome changes, namely aneuploidy, CNV, and LOH.

CNVs frequently arise under environmental stress and are caused by both DNA replication– and recombination–based mechanisms. CNVs can arise via intrachromosomal or interchromosomal events, and the frequency of each type depends on the underlying mechanism. Many CNVs, including deletions and duplications, arise via nonallelic homologous recombination between repeat sequences (also known as ectopic recombination) (21, 99). For example, recombination between transposable elements can generate CNVs in the plant pathogen *Zymoseptoria tritici*, and the stress from infection process can drive transposon mobilization and subsequent CNV formation (40, 41). A novel mechanism leading to high copy numbers (more than 12 copies) of large genomic regions occurs in *C. albicans* under the selection of antifungal drug stress. These high copy amplifications can arise via a dicentric chromosome intermediate and successive breakage–fusion–bridge cycles that are repaired by nonallelic homologous recombination between long repeat sequences, resulting in an accordion-like expansion of chromosomal DNA (128). High copy number CNVs can also be present in the form of extrachromosomal circular DNA (eccDNA) that arises via intrachromosomal nonallelic homologous recombination and is excised from the chromosome, similar to eccDNA in some human cancer cells (80). In some cases, these circular fragments can replicate autonomously. One study identified eccDNA covering more than 20% of the total yeast genome present in a population of yeast deletion mutants (81).

DNA replication–based mechanisms also result in CNV formation and are likely an underappreciated source of de novo CNVs in *S. cerevisiae* and other fungi. Replication-based mechanisms include the origin-dependent inverted-repeat amplification (13) and microhomology-mediated break–induced replication (52, 146). Additionally, in *S. cerevisiae* histone acetylation (H3K56ac) can promote CNV formation via repeated fork stalling or template switching, resulting in a higher copy number of genes that are highly transcribed in a stressful environment, like the copper transporter *CUP1* in copper-rich environments (60). These replication-based mechanisms suggest that CNV formation may not be random across the genome but rather that CNVs can increase in response to environmental stimuli because increased transcription may increase the frequency of collisions between the replisome and transcriptional machinery, causing an increase in the frequency of DNA damage and aberrant repair that in turn results in CNV.

## GENOMIC REARRANGEMENTS DURING ADAPTATION TO NEW ENVIRONMENTS

Stress from both environmental and host factors is believed to promote fungal genomic rearrangements. There are many examples of environmental stress that can promote genome rearrangements, including heat shock, nutrient limitation, hypoxia, and oxidative stress (11, 34, 82). For *S. cerevisiae*, heat shock can inhibit the function of heat shock protein 90 (Hsp90), causing proteotoxic stress and an increase in chromosome missegregation (19). Over longer timescales, adaptation to heat stress selects for recurrent aneuploid karyotypes (143). Fungal cells frequently encounter nutrient limitations in their environment that select for extensive genetic variations like CNVs. Selective pressures that strains routinely experience might explain the association of widespread large structural rearrangements and CNVs in wine strains of *S. cerevisiae* that are exposed to high ethanol and fermentation environments (123). These CNVs contained genes involved in the fermentation process, which includes flocculation, glucose metabolism, maltose metabolism, and copper resistance (123). Finally, while most examples of genomic rearrangement during adaptation to new environments involve amplification via aneuploidy or CNV, examples of chromosome loss also occur; for example, growth on the carbon source l-sorbose selects for monosomy of chromosome 5 in *C. albicans* (63).

Fungal cells are also exposed to complex stress environments within their hosts that can induce genomic rearrangements. In *C. albicans*, a single pass through a mouse host results in an increased frequency of aneuploidy and long-tract LOH events compared with in vitro–evolved populations (36–38). This finding indicates that genomic changes happen frequently during adaptation to host-related stresses in vivo. Oxidative stress is one of the primary host immune stresses and has a strong impact on fungal genome stability. When exposed to hydrogen peroxide, *C. albicans* exhibits a 40-fold increase in LOH relative to nonstress conditions (34). In *C. neoformans*, host-derived nitrosative stress directly contributes to the formation of larger titan (polyploid) cells by increasing superoxide production (148).

A high frequency of ploidy variation and aneuploidy is also observed in clinical isolates of diverse species, suggesting these genomic changes may be caused by or selected for during interaction with the host (16, 55, 150). *S. cerevisiae* is rarely found to cause invasive fungal infections; however, polyploidy is common across 132 clinical isolates, with 34% identified as triploid (3N) or tetraploid (4N). In addition, ~36% of these clinical isolates were aneuploid, and ~24% of strains exhibited CNVs (150). In clinical isolates of *C. albicans*, genetic variation is also evident at the individual-chromosome level. Multiple isolates contain large-scale CNVs (8/21) and aneuploidy (6/21) for the shortest chromosomes 4–7 (55). These data demonstrate that large genomic changes are an important part of the genomic architecture of fungal clinical isolates and highlight the potential connection between large-scale genomic changes and adaptation to a host environment.

## GENOME REARRANGEMENTS DURING ANTIFUNGAL DRUG TREATMENT

Antifungal drugs including azoles, polyenes, echinocandins, and 5-fluorocytosine are commonly used to treat human fungal infections (32, 102). Most of these antifungal drugs are fungistatic, not fungicidal, enabling some fungal growth and drug resistance to arise. The fungistatic azole drug fluconazole is widely prescribed yet can lead to polyploidy, aneuploidy, and CNVs that cause azole resistance (8, 112, 114). Adaptation to fluconazole via changes in copy number has been observed across diverse fungal species. Remarkably, the genomic change in a majority of these examples is the same: an amplification of the chromosome or chromosome region containing the drug target, encoded by *ERG11*. This amplification was first observed in *C. albicans*, which rapidly amplifies the left arm of chromosome 5 in an isochromosome structure during adaptation to fluconazole in vitro and in vivo (112, 128, 129). Isochromosome 5L causes multiazole drug resistance via amplification of two genes in a copy-dependent manner; these genes, *ERG11* and *TAC1*, encode the drug target and a transcriptional regulator of drug efflux pumps, respectively (113). Similarly, *C. glabrata*, *C. auris*, and *C. neoformans* can adapt to fluconazole in vitro and in vivo by acquisition of an aneuploidy or CNV of the chromosome that contains *ERG11* and often several other drug resistance genes like *TAC1* or drug efflux pumps (9, 15, 65, 119, 120, 140). Exposure to fluconazole can also promote adaptation to other drug classes due to the acquisition of different aneuploid chromosomes. For example, *C. neoformans* aneuploid strains that arose during adaptation to fluconazole also acquired cross-tolerance to all three classes of antifungal drugs (azole, polyenes, and 5-fluorocytosine) used for cryptococcosis treatment (140).

Exposure to antifungal drug stress can significantly increase the rate of genome rearrangements, yet the mechanisms driving this increase are not fully understood. In *C. neoformans*, fluconazole can bind with metal, generating increased intracellular reactive oxygen species, and higher levels of reactive oxygen species can directly lead to increased DNA damage and subsequent chromosomal instability (96). In *C. albicans*, fluconazole treatment disrupts kinetochore function by depleting the centromeric histone H3 variant (CENP-A/Cse4) and causing increased chromosome nondisjunction events (14). Depletion of histone H3 during fluconazole treatment may also promote the formation of dicentric chromosomes that are unstable and undergo recombination events that promote CNV and LOH as described above (112, 113, 128).

## THE EFFECTS OF POLYPLOIDY, ANEUPLOIDY, AND COPY NUMBER VARIATION ON FITNESS

Polyploidy, aneuploidy, and CNVs have a wide variety of effects on cellular fitness that are often environment specific. The fitness effects of these genomic changes are of great interest because fitness will ultimately determine whether they are fixed, are removed, or persist as low-frequency standing genetic variation within a fungal population, as well as the rate at which any of these fates will occur. The fitness effects of aneuploidy and CNVs, whether positive or negative, are the result of changes in gene expression for genes experiencing copy number changes and are also highly context and background specific.

Aneuploidies, and to a lesser extent CNVs, generally have a negative fitness effect in *C. albicans*, *S. cerevisiae*, and *Schizosaccharomyces pombe* when grown in complete laboratory medium (55, 87, 94, 131, 142, 151). The fitness costs associated with aneuploidy are thought to arise from increased protein abundance, resulting in hypo-osmotic stress (131, 134). Fitness costs in complete medium are less severe when only a portion of a chromosome is amplified or deleted. CNVs with minimal fitness costs have been observed in clinical isolates of *C. albicans* (114, 129) and in *S. cerevisiae* strains bearing artificially generated CNVs (86). In the latter study, across a panel of 53 strains bearing different CNVs, all but 2 strains showed growth comparable to wild type when grown in complete medium (86). Thus, CNVs appear to generally have lower fitness costs than full-chromosome aneuploidies. Although a general fitness cost would lead one to believe that aneuploidies would be rarely recovered in natural populations, they are frequently identified in clinical and environmental fungal isolates, as described above. In addition, they are frequently recovered from experimental evolution studies in multiple fungal species (4, 28, 34, 114, 119, 126, 141).

The frequent recovery of clinical and environmental isolates containing aneuploidies suggests that the fitness cost of aneuploidy can be negated by the fitness advantages induced by specific rearrangements in an environment-specific manner. In fact, natural aneuploid or CNV-bearing strains could be even more common than current sampling suggests, as they might be lost when cultivated outside of a selective environment where they are unstable, preventing their detection (42, 128, 141). Genetic background also plays a major role in the fitness effects of aneuploidies, as significant variation in tolerance to aneuploidy exists across genetically diverse strains and species of *S. cerevisiae* (67). A study looking for patterns in aneuploidy prevalence across 1,000 *S. cerevisiae* strains found that aneuploidy prevalence was explained better by genotype than by environment (109), highlighting the importance of genetic background in aneuploidy tolerance. In fact, the higher stability and minimal growth defects of wild yeast aneuploid strains in comparison to laboratory strains engineered to contain aneuploid chromosomes enabled the identification of genetic variation at the *SSD1* locus that provides increased tolerance of aneuploidy in wild yeast aneuploids compared with laboratory strain backgrounds (57, 58). In addition, organisms with higher ploidy levels (diploids and polyploids) have lower fitness defects when they are aneuploid (58, 115), possibly because aneuploidies result in a smaller proportional increase in total genomic content. As new methods to detect structural variation directly from short read sequencing of environmental samples are developed, the frequency of structural variants discovered in natural populations is almost guaranteed to rise.

## MECHANISMS OF ADAPTATION VIA ANEUPLOIDY AND COPY NUMBER VARIATION

There are a growing number of documented cases in which aneuploidies or CNVs confer a fitness advantage in a specific environmental condition. Phenotypes such as the ability to grow at low nutrient conditions (48, 60, 95), increased freeze–thaw tolerance (139), and changes in virulence and filamentation (38, 39, 54) have all been observed in isolates bearing aneuploidies or CNVs. In a genome-wide association study analyzing more than 1,000 isolates of *S. cerevisiae*, CNVs explain a larger proportion of variance in traits such as growth at high temperature than do single-nucleotide polymorphisms (97). In some cases, the specific mechanisms of phenotypic change have been uncovered. For example, in *Z. tritici*, multiple instances of insertion of a transposable element lead to overexpression of multidrug resistance genes (78, 90, 91), and multiple instances of ectopic recombination between TEs lead to the deletion of an effector gene that allowed the fungus to escape detection by the host plant (51). Experimental evolution studies have also shown that aneuploidy and CNVs can provide a rapid mechanism of adaptation to genetic defects. In one remarkable example of adaptation to an extreme genetic defect—loss of a myosin required for cytokinesis—*S. cerevisiae* strains adapted via amplification of a transcription factor, *RLM1*, regulating processes in cell wall remodeling (101). This example is also not an outlier, as a large-scale screen for adaptive evolution of supposedly essential gene deletions found frequent adaptation via ploidy changes and acquisition of aneuploidy (74).

In the case of copy number increases, fitness advantages are typically the result of increases in the abundance of a protein encoded by a gene within the amplified region that is required for growth in that environmental condition. There are multiple examples of experimental evolution in *S. cerevisiae* and closely related species in low-nutrient conditions that resulted in recurrent amplifications of genomic regions specific to the growth condition. While the exact region amplified varied between evolved isolates, amplifications were repeatedly observed that contained the genes encoding glucose transporters *HXT6* and *HXT7* in a low-glucose environment, the sulfur transporters *SUL1* and *SUL2* in sulfate-limiting conditions, the phosphatase *PHO5* in a phosphate-limiting environment, *GAP1* in nitrogen-limiting environments, and the copper transporter *CUP1* in a copper-rich environment (47, 48, 60, 69, 105). Clearly, amplifications can be advantageous when they result in an increase in copy number for a gene that limits growth in a particular environment. Fitness advantages are often specific to the environmental stressor, and the same CNVs or aneuploidies that are selected for in a particular environment often have negative fitness consequences when the stress is removed.

**Figure 2:**
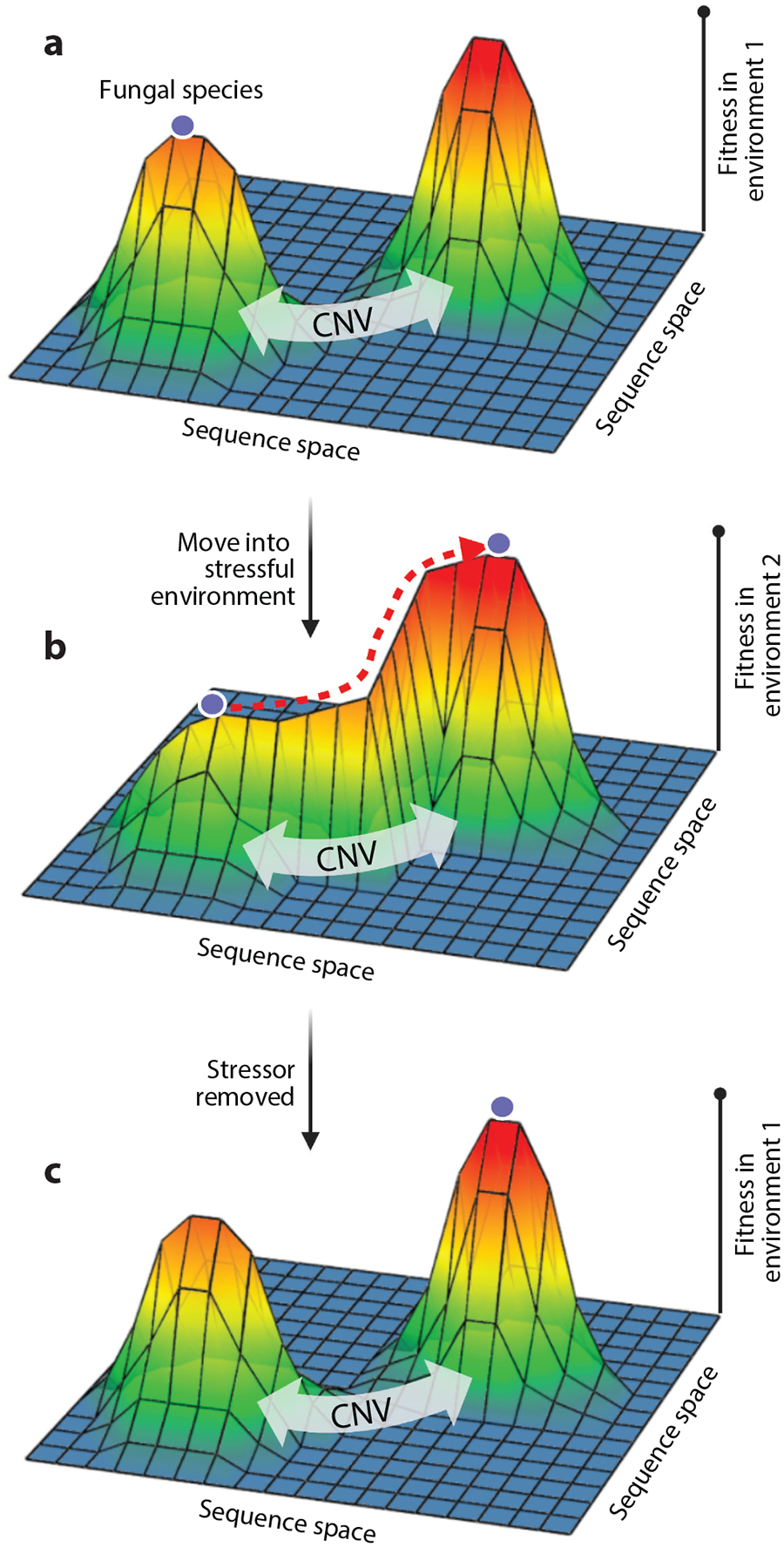
(*a*) The fitness landscape in one environment may prevent a stepwise evolutionary trajectory of a fungal species (*purple circle*) toward another genotypic space with high fitness in multiple environments (*right fitness peak*). (*b*) Copy number variations (CNVs) can have higher fitness in stressful environments than in a nonstressful environment, providing (*c*) a bridge to the genotypic space with high fitness in both stressful and nonstressful environments. Fitness landscapes adapted with permission from Reference 127. Figure created in BioRender.com.

The environmental specificity of fitness advantages of aneuploidies and CNVs suggests that they represent a temporary adaptation, allowing cells to survive long enough in the stressful environment to acquire new, less costly solutions to the challenge that will persist after the stressor is removed, thereby serving as bridges between peaks on the fitness landscape (33, 93, 133) (Figure 2). An experimental evolution study in *S. cerevisiae* found recurrent trisomy of chromosome 3 in response to heat stress that was later replaced by gene-level modifications, although the specific modifications were not identified (143). Examples of aneuploidies or CNVs providing a temporary solution to an environmental challenge that is subsequently replaced by point mutations in fungal species are sparse, but there are some remarkable studies in other kingdoms that can serve as a proof of principle. For example, experimental evolution for drug resistance in the bacterium *Salmonella* initially resulted in amplification of the β-lactam gene conferring a degree of resistance, which was then replaced by other mutations elsewhere in the genome that conferred higher fitness in subsequent passages (125). Clinical isolates of *C. albicans* in which aneuploidies are initially detected but are not found in subsequent serial isolates from the same patient suggest that aneuploidies or CNVs might serve as bridges between fitness peaks in the host (39).

In addition to providing more time for another mutation to arise, increases in copy number provide an increased target size for beneficial mutations to occur on the amplified genes themselves. Once again, while examples from within the fungal kingdom are lacking, a striking example of just such an occurrence comes from the experimental evolution of poxvirus to evade host defense systems. In just 10 passages, a key enzyme counteracting host defense was amplified up to 4 times. One of the copies acquired a point mutation that increased enzymatic efficiency, after which the additional copies of the gene were lost (31). Similar expansions in copy number of genomic regions have been observed in *C. albicans*, although the acquisition of point mutations in these regions has not yet been observed (128). Evolution experiments have also shown that strains of *S. cerevisiae* with higher ploidy diverge and adapt more rapidly than those with lower ploidy (101, 115), and cells with higher ploidy explore a larger range of adaptive mutations (110).

The increased rates and number of adaptive routes that aneuploidy provides for fungal cells are also true of cancer cells, making fungi a good model system for combating adaptation in tumors. Increased genomic instability that results from unique karyotypes has been observed in yeast (118) and in cancer cells, driving attempts to target CNV itself as a method of preventing tumor proliferation and resistance to chemotherapy (61, 77, 149). If increased copy number does act as a bridge to other adaptations, sensitizing cells to aneuploidy may prevent quick and easily accessible routes of adaptation in cases where it is not desired. For example, an *S. cerevisiae* population with heterogeneous aneuploid genotype was found to have a wider range of phenotypes and a greater ability to acquire resistance than a homogeneous euploid population. To combat the acquisition of resistance, researchers identified compounds that would first channel evolving populations to a specific aneuploid genotype and then target the susceptibility of that specific aneuploid genotype (20). This elegant “evolutionary trap” method could be useful in combating the evolution of drug resistance in fungi and human cancer cells.

## THE EFFECTS OF ANEUPLOIDY AND COPY NUMBER VARIATION ON GENE EXPRESSION

**Figure 3:**
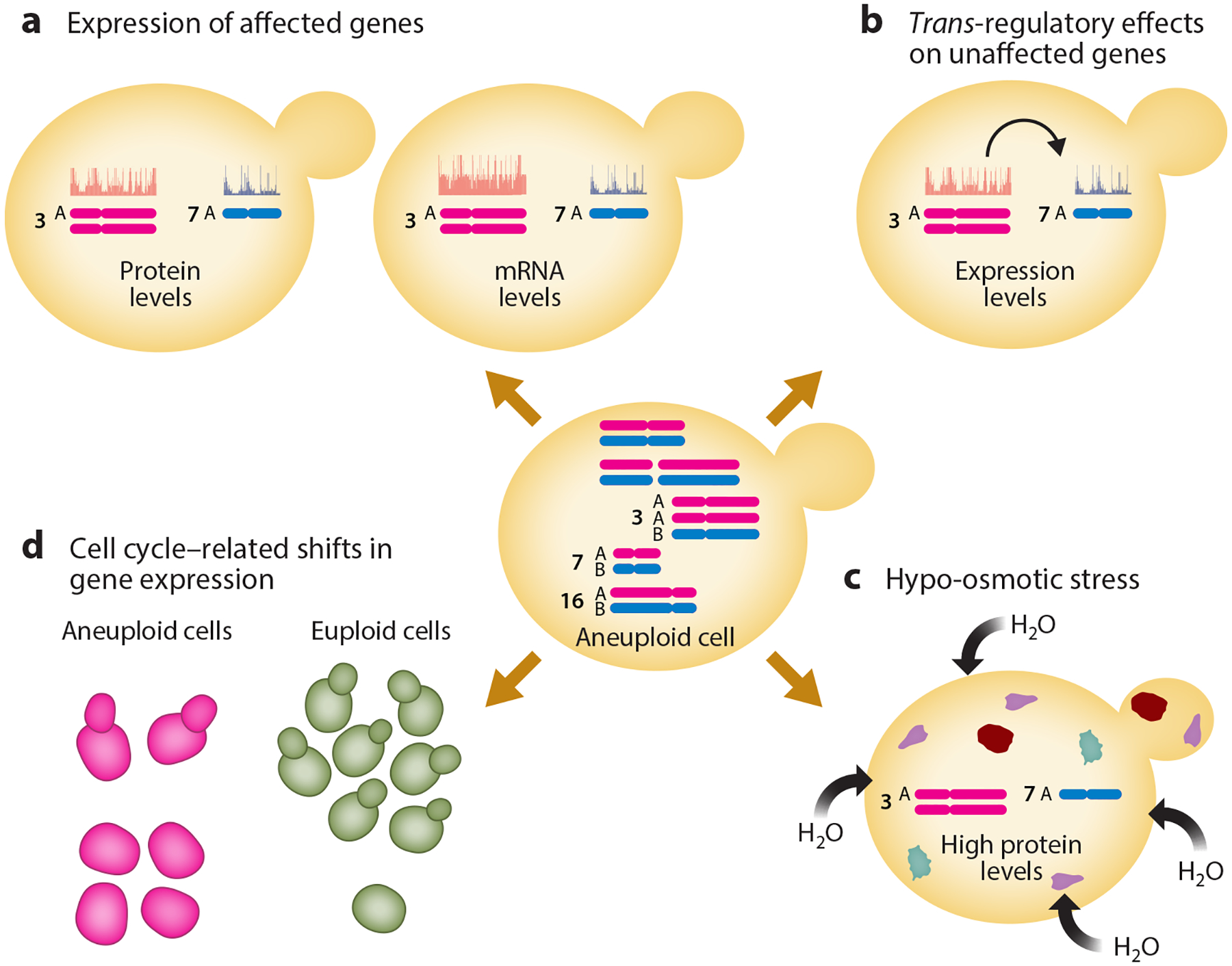
Changes in gene expression detected in aneuploid cells or cells bearing copy number variations are the result of multiple factors. These factors include (*a*) the change in copy number of the gene itself, (*b*) *trans-*regulatory effects of amplified genes on expression of other genes across the genome, (*c*) hypo-osmotic stress or other aneuploidy-related stresses inducing gene expression changes, and (*d*) shifts in the proportion of cells in particular cell cycle phases that in turn result in changes in the representation of certain transcripts in nonsynchronized bulk samples. Figure adapted from images created in BioRender.com.

Aneuploidy and CNV can result in widespread changes to gene expression throughout the genome. The total set of gene expression changes observed in a cell that contains an aneuploidy or CNV is the result of multiple factors. First, changes in copy number often result in the differential expression of the genes with increased or decreased copy number or whose regulatory regions have been influenced by an insertion or deletion. The degree to which dosage compensation may reduce over- or underexpression of genes on aneuploid chromosomes continues to be debated and appears to be background specific. Second, differential expression of genes that have undergone a copy number change have *trans*-regulatory effects on expression of genes elsewhere in the genome. Third, gene expression changes can occur that are associated with cellular stress resulting from the increased transcription and translation of amplified genes and/or additional genomic material. Finally, some changes in gene expression are the result of altered growth of the strain bearing the aneuploidy (106) (Figure 3). Much research evaluating the consequences of aneuploidy on gene expression, which was originally done in a limited number of laboratory strains, is now being performed across a wider range of isolates and genetic backgrounds. These additional studies demonstrate that genetic background has a strong impact on the consequences of aneuploidy and CNV.

Gene expression levels of the genes encoded on an aneuploid chromosome are on average increased or decreased proportionally to DNA copy number (59, 101, 110, 112, 124, 131). Increases in the number of transcripts produced from each gene on an aneuploid chromosome result in an increase in the total number of proteins produced (94). The upregulation of many genes simultaneously can result in adaptation to multiple stressors at once, such as in the evolution of multidrug resistance (141). Although the overexpression of certain genes on an aneuploid chromosome can be advantageous in some environments, the general overexpression of proteins will lead to fitness defects that are related to the total higher abundance of proteins in the cell, resulting in proteotoxic or osmotic stress (68, 94, 131, 134, 138). The fitness cost of overexpression could contribute to the fact that smaller chromosomes that bear fewer genes are more frequently aneuploid in natural isolates across multiple species (46). Furthermore, in *S. cerevisiae*, the proliferation rate is negatively correlated with the number of open reading frames encoded on additional chromosomes (117, 131), and larger chromosomes are more frequently lost than smaller chromosomes after whole-genome duplication events (66).

Over- or underexpression of genes that encode one component of a larger protein complex can also be a source of fitness defects in aneuploid cells. Proteins can become cytotoxic when their stoichiometry relative to the rest of the complex is off balance (10, 64, 92, 122, 137). In this case, negative fitness consequences come from imbalanced protein complexes, and changes in copy number that maintain a complex’s stoichiometry should have lower fitness costs. These stoichiometric relationships could contribute to the recurrent patterns of chromosome loss observed in tetraploid strains of *S. cerevisiae* that suggest there is selection for specific copy number relationships between chromosomes (115). However, recent studies in cells containing multiple aneuploidies have shown that protein complexes in which all members of the complex are balanced remain overexpressed, while imbalanced complexes tend to be degraded and restored to a typical copy number by some form of dosage compensation. In at least these cases, the overexpressed balanced protein complexes tend to be the ones inhibiting proliferation (22). Indeed, studies have observed in human cells and natural isolates of *S. cerevisiae* that genes involved in large complexes (83, 94, 108, 124) and under strong constraints on expression level (58) are more likely to be subject to dosage compensation, in which feedback mechanisms in the cell reduce expression levels closer to those of wild type even when they have been amplified. How aneuploidy and CNVs affect the stoichiometry of large protein complex members is likely dependent on the level to which dosage compensation occurs after copy number amplification.

## DOSAGE COMPENSATION IN ANEUPLOID CELLS

Dosage compensation mechanisms that reduce overexpression of individual genes on aneuploid chromosomes appear to frequently operate at the protein level rather than, or in addition to, the transcript level. In *C. albicans* as well as in human cells, mRNA levels are directly proportional to DNA copy number, but protein levels are not as highly overexpressed (83, 108, 124). Buffering of overexpression at the posttranslational level can minimize the fitness cost of an aneuploidy. For example, mutations disrupting the deubiquitinizing enzyme *UBP6* increased the growth rates of various aneuploid strains of *S. cerevisiae* (130), likely by increasing protein degradation rates and bringing total protein abundance closer to that of a euploid strain. Likewise, aneuploid wild isolates of *S. cerevisiae* that showed evidence of dosage compensation at genes with highly constrained expression levels had much smaller fitness defects than laboratory strains that did not show dosage compensation (58). As mentioned above, differences in dosage compensation and tolerance to aneuploidy between the commonly used lab strain W303 and a wild isolate of *S. cerevisiae* were mapped to genetic variation at the *SSD1* locus, which is an RNA-binding translational regulator (57). Thus, it is becoming increasingly apparent that, when investigating buffering of gene expression and variability in the fitness costs of CNV, it is important to assay a diversity of genetic backgrounds. In addition, the strong effect of *SSD1*, a translational regulator, highlights the importance of assaying not only transcript levels but also protein levels.

The fitness costs of changes in gene expression vary from gene to gene. Variability in dosage sensitivity across the genome has long been appreciated from large-scale surveys assaying the fitness consequences of overexpression of single genes (18, 25, 75, 103, 121) or of underexpression in heterozygous null strains (92). Less well appreciated, however, is the fact that dosage sensitivity, with or without any sort of dosage compensation for changing copy number, is also species and strain specific (58, 68, 103, 109). For example, a study examining the fitness effects of more than 4,000 genes individually overexpressed in 15 different lineages of *S. cerevisiae* found that the fitness costs of overexpression were dominated by strain-specific effects (103).

## *TRANS*-REGULATORY EFFECTS OF ANEUPLOIDY AND COPY NUMBER VARIATION

When genes encoded on an aneuploid chromosome are differentially expressed, they can have *trans*-regulatory effects that lead to differential expression of genes located elsewhere in the genome. Overexpression of transcription factors in *S. cerevisiae* results in the upregulation of their downstream targets elsewhere in the genome (47, 101, 121), and indirect *trans*-regulatory effects also occur at genes other than transcription factors and their targets (136). In fact, *trans-*regulatory changes account for most variation in gene expression between strains of *S. cerevisiae* (2), highlighting the importance of *trans*-acting factors for gene expression divergence within species. However, more work is needed to understand the extent to which copy number changes contribute to *trans*-regulatory divergence within fungal species.

## TRANSCRIPTOMIC SIGNATURES OF STRESS INDUCED BY ANEUPLOIDY

Aneuploid cells can also have a gene expression signature that is common across slow-growing cells. These gene expression changes are the consequence of changes in progression through the cell cycle, influencing the proportion of cells in a clonal population that are currently in specific phases of the cell cycle. This recurrent set of transcriptomic changes has been termed a transcriptomic slow growth signature. A slow growth signature resembles the general environmental stress response gene expression signature and can also be understood as a general transcriptional readout of stressed cells (43, 44, 88). The gene expression changes that compose this transcriptional signature can be informative as to whether cells are experiencing stress and slowing their progression through the cell cycle but can also constitute noise in gene expression data that must be removed to reveal gene expression changes that are specific to a particular aneuploidy or CNV (88). Different approaches to minimize or remove slow growth or stress response signatures from gene expression data have been employed. One such approach is to experimentally manipulate environmental growth conditions until the growth rates of euploid and aneuploid cells are matched before collecting RNA for sequencing (131). Another approach is to mathematically subtract the gene expression changes that correlate highly with growth rate across diverse perturbations (88). How these general gene expression signatures of slow growth and stress are treated will depend upon the goals of the study, as well as the experimental options available. Regardless of the specific application, it is important to be aware of the potential presence of a slow growth signature in gene expression data.

## OUTLOOK AND FUTURE PERSPECTIVES

The need to understand the dynamic fungal genome is apparent, but much work remains to be done to fully understand the mechanisms and effects of large genomic changes so that these changes may be predicted, promoted, or combated. One of the challenges of studying CNVs is their transient nature, which often results in the development of heterogeneous populations even when starting with a single clone (128). The advent of single-cell sorting and single-cell sequencing and their adaptation to fungi specifically (62, 69, 79, 135) provides a new avenue to assess colony heterogeneity in copy number. Although the fungal cell wall can make single-cell technologies such as microfluidics-based droplet sequencing challenging, protocols and new methodologies are being worked out to meet this challenge (27).

In addition to a better understanding of population heterogeneity, more research is needed to address the mechanisms of formation of various types of genomic rearrangements. Understanding the mechanisms underlying large genomic changes will help predict frequency under different stress conditions, such as the different antifungal drug concentrations that are encountered in the human host. In addition, findings in fungi can be applied to other cell types that frequently experience large genomic changes, such as cancer cells, in which whole-genome duplications and aneuploidies are associated with increased tumor growth and resistance to chemotherapies (61, 100). Continued progress in understanding the mechanisms and rates of formation in conjunction with a better understanding of their phenotypic consequences will allow a better prediction of the arrival and survival of ploidy changes, aneuploidy, CNVs, and LOH in fungal populations and beyond.

The difficulty of developing new antifungal drugs and agricultural fungicides makes it crucial that we understand the mechanisms by which resistance develops to our limited arsenal of drugs. The incredibly dynamic fungal genome is a major pathway to resistance for multiple species of clinical and agricultural importance. Establishing the link between ploidy, aneuploidy, and copy number changes and fungal adaptation is an important first step, but more work must be done to increase our power to predict these genomic changes and uncover their susceptibilities.

## Abbreviations

Ploidy: the number of complete sets of chromosomes in a cell, usually one or two in fungi. Sometimes individuals from the same species can exist as stable haploid or diploid cells
Aneuploidy: an abnormal chromosome number; can include copy number gain or loss of an entire chromosome
Copy number variation (CNV): amplification or deletion of a chromosomal segment; may include small or large amplification/deletion events and may amplify one or many extra copies
Polyploid: describes an organism with more than two complete sets of chromosomes; can be due to whole-genome duplication or hybridization events, both ancient and recent
Loss of heterozygosity (LOH): genetic variation that occurs in diploid fungi, resulting in the loss of one of one haplotype; may be caused by gene conversion, break-induced replication, or whole-chromosome loss
Euploid: describes an organism with a complete set of chromosomes
